# Acute deoxygenation during M-TEER: a note of caution

**DOI:** 10.1093/ehjimp/qyag171

**Published:** 2026-10-06

**Authors:** Michael Wester, Michael Paulus, Andreas Luchner, Christian Schach, Bernhard Unsöld, Lars S Maier, Can Martin Sag, Christine Meindl, Kurt Debl

**Affiliations:** University Heart Centre Regensburg, Franz-Josef-Strauß-Allee 11, Regensburg 93053, Germany; University Heart Centre Regensburg, Franz-Josef-Strauß-Allee 11, Regensburg 93053, Germany; Krankenhaus Barmherzige Brüder Regensburg, Regensburg, Germany; University Heart Centre Regensburg, Franz-Josef-Strauß-Allee 11, Regensburg 93053, Germany; University Heart Centre Regensburg, Franz-Josef-Strauß-Allee 11, Regensburg 93053, Germany; Medical Clinic I, Cardiology and Angiology, Justus-Liebig-University, Giessen, Germany; University Heart Centre Regensburg, Franz-Josef-Strauß-Allee 11, Regensburg 93053, Germany; University Heart Centre Regensburg, Franz-Josef-Strauß-Allee 11, Regensburg 93053, Germany; University Heart Centre Regensburg, Franz-Josef-Strauß-Allee 11, Regensburg 93053, Germany; University Heart Centre Regensburg, Franz-Josef-Strauß-Allee 11, Regensburg 93053, Germany

## Abstract

**Aims:**

Right-to-left (R-L) shunting through an iatrogenic atrial septal defect (iASD) after mitral transcatheter edge-to-edge repair (M-TEER) is a rare but potentially life-threatening complication. We aimed to identify factors associated with clinically relevant R-L shunting requiring emergency iASD closure.

**Methods and results:**

We retrospectively analysed 444 consecutive patients undergoing successful M-TEER between 2017 and 2026. Clinical, echocardiographic, and invasive haemodynamic parameters were compared between patients requiring emergency iASD closure and the remaining cohort. Six patients (1.3%) required emergency iASD closure due to R-L shunting and acute deoxygenation. All presented with severe tricuspid regurgitation, RV dilatation, and impaired RV function. Following M-TEER, right atrial pressure exceeded left atrial pressure in five patients. Compared with controls, affected patients demonstrated reduced tricuspid annular plane systolic excursion (15 ± 2 vs. 19 ± 4 mm), fractional area change (FAC) (27 ± 5% vs. 43 ± 6%), and RV free wall strain (−13 ± 2% vs. −22 ± 5%). Echocardiography underestimated invasively measured systolic pulmonary artery pressure (25.8 vs. 40.3 mmHg; *P* = 0.035). Oxygen saturation decreased by 3-8%, with one patient developing cardiogenic shock.

**Conclusion:**

Severe tricuspid regurgitation, RV dysfunction, and elevated right atrial pressure may identify patients at risk for clinically relevant R-L shunting after M-TEER. Comprehensive RV assessment, including FAC and strain, combined with invasive hemodynamic evaluation, may improve risk stratification and facilitate timely iASD closure.

## Graphical Abstract

**Graphical Abstract:**
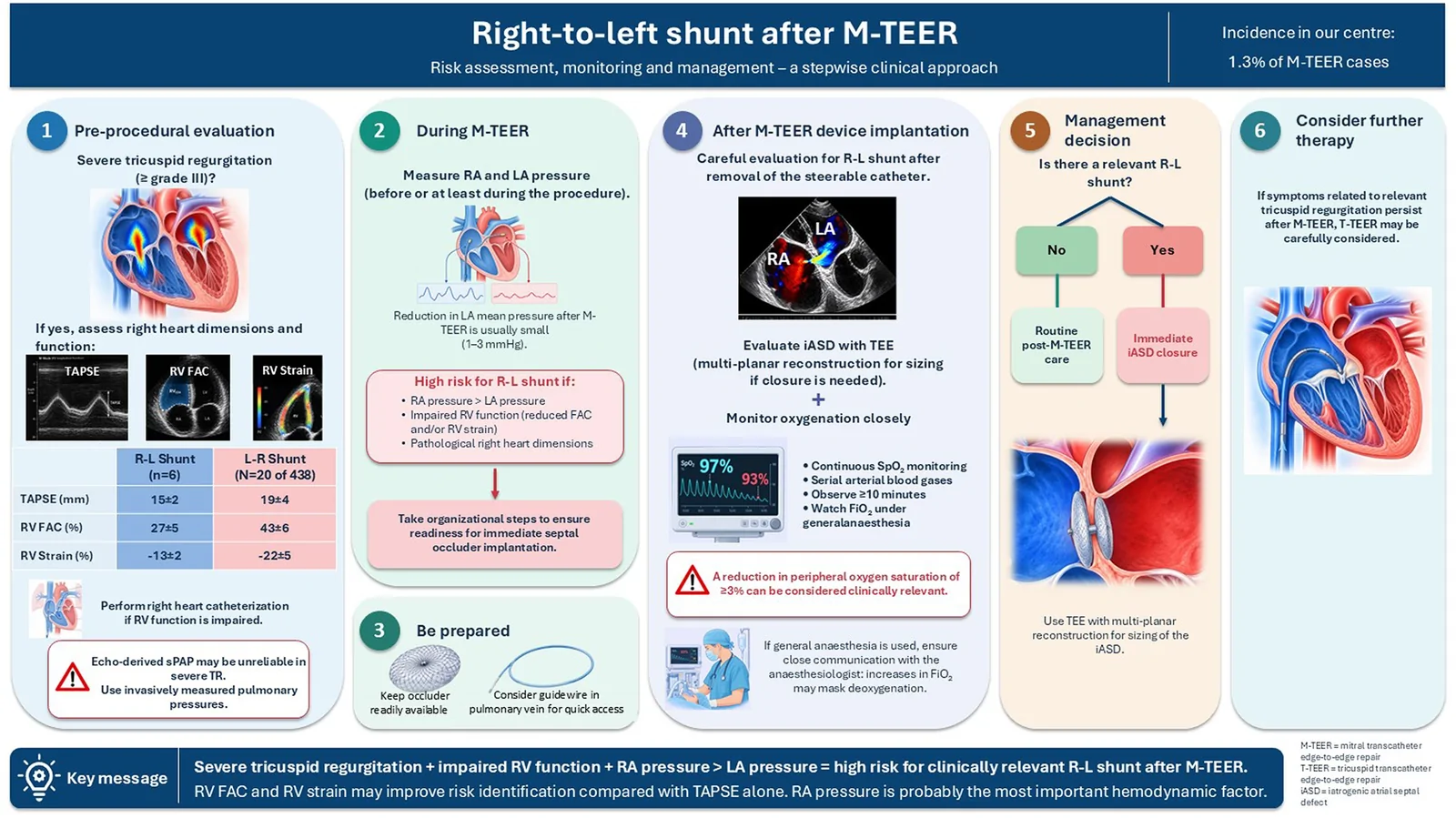

## Introduction

M-TEER has become a standard therapy option for patients with symptomatic severe mitral regurgitation.^1^ Device implantation is performed safely with good procedural and symptomatic outcomes.^1^ The transseptal puncture with a large bore catheter necessary for device implantation causes an iatrogenic atrial septal defect (iASD) which often persists after the procedure. The prevalence after 1 month is 43–81% and after 1 year 27–42%.^2^ The haemodynamic effect of this small iASD is often negligible; however, in some cases, iASD closure becomes necessary. This is mostly due to a relevant left-to-right shunt with subsequently resulting right heart overload and right heart failure.^3^ However, in rare cases, the iASD leads to a relevant right-to-left (R–L) shunt causing systemic deoxygenation. Morikawa *et al.*^4^ provided a large single-centre cohort of 385 consecutive M-TEER patients in which R–L shunt occurred in 20 patients (5.2%). Of these, acute deoxygenation was present in seven cases, necessitating prompt iASD closure (1.8%).^4^ R–L shunting thus poses an acute emergency and organizational preparation for immediate iASD closure is essential. Therefore, predictors for R–L shunt are crucial. Morikawa identified the presence of severe tricuspid regurgitation, and higher right atrial pressure as predictors for R–L shunt.^4^ A large survey included all M-TEER patients in Japan treated with M-TEER from April 2018 to November 2020 totalling 2722 patients. Of these, 30 (1.1%) required iASD closure and 13 (0.4%) due to hypoxemia with R–L shunt.^5^ The authors do not provide criteria that would allow the identification of predictors for R–L shunt or necessity for iASD closure. This highlights that significant R–L shunt after M-TEER is a rare but serious adverse event requiring immediate iASD closure. Reported case numbers are still very low, and more data are needed to guide clinical decision-making.

The aim of this analysis is to identify factors that are associated with the occurrence of relevant R–L shunt with acute deoxygenation after M-TEER necessitating prompt iASD closure.

## Methods

### Study population

We retrospectively reviewed 444 patients with grade ≥III primary or secondary mitral regurgitation who were considered high risk for surgery and who underwent successful M-TEER at the University Heart Centre Regensburg, Regensburg, Germany from September 2017 to April 2026. We report six patients in whom emergency iASD closure after removal of the transseptal catheter was necessary due to acute deoxygenation. We show data from the whole M-TEER patient cohort as a comparison to identify risk factors for R–L shunt. As we do not routinely measure fractional area change (FAC) and RV free wall strain, we randomly selected 20 patients out of the total cohort and analysed FAC and RV free wall strain in this sub-cohort. There was no significant difference between this subgroup and the total cohort [age, BMI, NT-pro-BNP, pre- or post-interventional LA mean pressure, pre- or post-interventional LA v-wave, LV ejection fraction, tricuspid annular plane systolic excursion (TAPSE), systolic pulmonary artery pressure (sPAP), right atrial (RA) area, right ventricular end-diastolic diameter (RVEDD), left atrial volumen index]. All patients gave written informed consent for the procedure and for inclusion in the RETORT-MR registry, which is approved by the local ethics committee (approval Z-2017-0862-10).

### M-TEER procedure

Patients were placed under general anaesthesia, and intra-procedural imaging was carried out using fluoroscopy in combination with transoesophageal echocardiography (TEE). Subsequently, TEE-guided puncture of the atrial septum was carried out using an 18-gauge Brockenbrough needle. Following this, an 8.5-F sheath was advanced into the left atrium.

Unfractionated heparin was administered to achieve sufficient systemic anticoagulation, with the activated clotting time monitored at regular intervals to maintain values above 300 s. A 0.035-inch Amplatzer Super Stiff wire (Boston Scientific, Marlborough, Massachusetts) was then advanced through the sheath and positioned within the left upper pulmonary vein.

Next, a large bore M-TEER steerable sheath was introduced into the left atrium. Baseline LA pressure measurements were obtained via the guide sheath catheter. The M-TEER procedure itself was subsequently performed according to the standard technique as previously described.

Upon completion of the intervention, repeat measurements of both left and right atrial pressures and v-waves were performed. At this stage, the iASD was evaluated using TEE.

### Echocardiography

Echocardiography images were retrospectively analysed for RV strain and FAC using QLab (V 15.6, Philips Ultrasound). In accordance with ASE guidelines, we report RV free wall strain.

During the M-TEER procedure, TEE was conducted and interpreted by the site’s echocardiographer. The direction of blood flow across the iASD was qualitatively evaluated using colour flow Doppler imaging. iASD size was quantified using 2D and 3D measurements.

### Statistical analysis

Due to the small number of patients with R–L shunt and iASD occluder, we have listed individual patient characteristics in *Table 1*. To allow for an informative comparison with M-TEER patients without R–L shunt and iASD occluder, we report the mean ± SD values for the parameters of the occluder patients and of the M-TEER patients without occluder and have performed Student’s *t*-test. This comparison should be cautiously interpreted given the small patient number. To give an outlook on possible echo parameters for RV function, we provide a comparison for TAPSE, RV strain, and FAC between patients with and without iASD occluder indication. As we do not routinely measure RV strain and FAC, we have randomly selected 20 patients from the 438 patients without iASD occlusion, and RV strain and FAC were retrospectively measured by an experienced blinded echocardiographer. Randomization was performed using the randomization tool in Microsoft Excel, and the first 20 patients with sufficient imaging of the right ventricle to perform RV strain and FAC were selected (the first 27 patients were analysed, and imaging quality was insufficient in 7 patients).

**Table 1:** Baseline characteristics of patients with relevant right-to-left shunting in comparison to patients without relevant shunting.

	Patient 1	Patient 2	Patient 3	Patient 4	Patient 5	Patient 6	Patients with R–L shunt Mean	Patients without R–L shunt Mean	*P*-value^a^
**Pre-procedural invasively measured haemodynamic parameters**									
Age, years	83	78	84	75	83	72	79.1 ± 4.9	76.4 ± 8.3	0.425
Sex	F	M	F	M	M	M			
Body mass index, kg/m^2^	28.1	19.9	24.3	30.0	24.8	25.1	25.3 ± 3.4	26.1 ± 5.1	0.686
NYHA class	III	IV	III	II	III	III			
Persistent atrial fibrillation	Yes	Yes	Paroxysmal	Yes	Yes	Yes			
Chronic obstructive pulmonary disease	No	No	No	No	No	No			
Creatinine, mg/dL	4.75	1.74	5.21	1.66	1.38	1.1	2.6 ± 1.8	1.7 ± 5.1	0.693
NT-pro-BNP, pg/dL	15 872	901	168 802	1409	1397	5090	32 245 ± 67 138	4013 ± 6082	**0**.**001**
Mean right atrial pressure, mmHg	28	19	11	20	22	17	19.5 ± 5.6		
Right atrial v-wave, mmHg	46	30	X	28	42	25	35.2 ± 10.3		
Pulmonary artery pressure, mmHg	46/25/32	34/14/22	51/17/28	41/15/24	39/15/22	31/11/20			
Pulmonary vascular resistance, wood units	5.4	1.3	X	2.2	1.8	2.8			
Mean left atrial pressure, mmHg	22	19	21	15	18	14	18.1 ± 3.1	16.7 ± 6.4	0.584
Left atrial v-wave, mmHg	26	23	43	25	28	22	27.8 ± 7.7	30.9 ± 15.1	0.616
**Pre-procedural echocardiographic parameters**									
Aetiology of mitral regurgitation	secondary	secondary	secondary	primary	secondary	secondary			
Mitral regurgitation grade	IV	IV	IV	IV	IV	IV			
Left ventricular ejection fraction, %	61%	60%	35%	56%	49%	47%	51.3 ± 9.8	47.7 ± 14.7	0.552
Tricuspid regurgitation grade	IV	IV	III	IV	IV	V			
TAPSE, mmHg	18	17	12	15	16	14	15.3 ± 2.1	19.0 ± 4.3	**0**.**035**
RV FAC, %	28%	33%	23%	27%	31%	20%	27.0 ± 4.9%	43.8 ± 6.1%^b^	**0**.**001**
RV free wall strain, %	−15.9%	−12.8%	−10.8%	−12.1%	−14.1%	−10%	−12.6 ± 2.2%	−22.2 ± 4.7%^b^	**0**.**001**
sPAP, mmHg	13	26	54	31	18	13	25.8 ± 15.5	43.0 ± 14.2	**0**.**004**
TAPSE/sPAP, mm/mmHg	1.38	0.65	0.22	0.48	0.89	1.08	0.59 ± 0.42	0.40 ± 0.17	**0**.**008**
RVEDD, mm	45	52	47	56	59	61	53.3 ± 6.4	39.6 ± 7.3	**0**.**001**
LA volume index, mL/m^2^	68	148	80	47	209	61	106.6 ± 69.5	81.0 ± 37.9	0.106
RA area, cm^2^	49	35	28	55	77	39	47.1 ± 17.5	27.6 ± 9.8	**0**.**001**
**Post-procedural invasively measured haemodynamic parameters**									
Mean left atrial pressure, mmHg	14	16	7	14	24	15	15.053.3 ± 5.4	14.9 ± 5.7	0.980
Left atrial v-wave, mmHg	16	20	10	22	40	18	21 ± 10.1		
Difference between mean RA and LA pressure, mmHg	14	3	4	6	−2	2	4.5 ± 5.4		
Shunt size, mm	14	7	10	8	6	7	8.7 ± 2.9		
Deoxygenation, SaO_2_%	99–60% and cardiogenic shock	97–94%	100–92%	97–91 mmHg	99–94%	97–92%			
iASD Device	Amplatzer Septal Occluder 19 mm	Amplatzer Septal Occluder 16 mm	Amplatzer Septal Occluder 16 mm	Amplatzer Septal Occluder 18 mm	Amplatzer Septal Occluder 18 mm	Amplatzer Septal Occluder 16 mm			
**Post-procedural echocardiographic parameters**									
Mitral regurgitation grade	II	I	I–II	I	II	I			
Transmitral gradient, mmHg	2	3	2	2	5	1	2.5 ± 1.3		
Tricuspid regurgitation grade	V	IV	III	IV	IV	V			

Bold values indicate statistical significance *p* < 0.05.

^a^ *P*-value for comparison of the mean for patients with R–L shunt and the mean for patients without R–L shunt.

^b^Subcohort of 20 randomly selected patients.

## Results

### Patient characteristics

From September 2017 to April 2026, we have performed 444 successful M-TEER procedures at our centre, of which 6 patients required immediate iASD occluder implantation, which equals 1.3% of all cases. Patient characteristics are presented in *Table 1*. The age of the six patients was 72–84 (79 ± 5) years; two were female and four were male. All had significant symptomatic mitral regurgitation. Left ventricular ejection fraction was normal in three patients, mildly reduced in two, and moderately reduced in one. LA and RA were severely dilated in all patients. All six patients had at least severe tricuspid regurgitation and reduced RV function and a dilated RV. Echo-derived sPAP was increased in only one patient; however, invasive measurement showed that pulmonary pressure was increased in all patients, and the lower echo-derived sPAP may be ascribed to the severe tricuspid regurgitation and functional loss of valve function and reduced RV function.

### Procedural characteristics

Successful M-TEER with effective reduction of mitral regurgitation was performed in all patients in this case series. The subsequent reduction of LA pressure led to higher RA pressures than LA pressures after device implantation, with a mean difference of 4.5 mmHg. LA pressure was higher than RA pressure in one patient (Patient 5), but the difference was only 2 mmHg. After removal of the steerable sheath from the interatrial septum, there was an immediate predominant R–L-shunt visible on TEE. The shunt size was 8 ± 3 mm (range 6–14 mm, derived from colour Doppler imaging). This resulted in a relevant deoxygenation ranging from a decrease in peripheral oxygen saturation (SpO_2_) of 3–8%. In one case (Patient 1), deoxygenation was life threatening, and immediate cardiogenic shock ensued with need for mechanical resuscitation and implantation of veno-arterial extracorporeal membrane oxygenation. The shunt was closed using an Amplatzer Septal Occluder in all patients (size 16–19 mm).

### Factors associated with relevant R–L shunt

A case series of six patients cannot reliably identify statistically significant predictors for relevant R–L shunt. However, it is striking that all patients in our series had severe tricuspid regurgitation, reduced right heart function (increased diameter; reduced systolic function [TAPSE, RV strain, RV FAC]). Invasive factors seem to be increased RA pressures and especially RA pressure exceeding post-procedural LA pressure. The comparison with the total M-TEER cohort without R–L shunt shows that right heart dimensions (RVEDD, RA area) were increased and RV function was decreased (TAPSE, FAC, RV free wall strain). Echo-derived sPAP was reduced in patients with R–L shunt. However, there was a relevant difference between echo-derived sPAP and invasively measured sPAP of 14 mmHg (25.8 vs. 40.3 mmHg, *P* = 0.035).

## Discussion

Our case series highlights several relevant aspects of M-TEER planning and intervention.

First, it shows that relevant R–L shunt is a rare (1.3% of the cases in our centre) but potentially very severe complication of M-TEER. Pre-procedural risk factors should be carefully evaluated. If a patient presents with concomitant severe tricuspid regurgitation, RV function should be carefully investigated, including RV strain and RV–PA coupling.

This is in accordance with the risk factors identified by Morikawa *et al.*, which were severe tricuspid regurgitation and right atrial pressure. Our patients all had RV dysfunction measured with TAPSE, RV FAC, and RV free wall strain. It is striking that TAPSE was only mildly reduced (15 ± 2 vs. 19 ± 4 mm, reference ≥17 mm) whereas RV FAC (27 ± 5 vs. 43 ± 6%; reference ≥35%) and RV free wall strain (−13 ± 2 vs. −22 ± 5%, reference <−20%) were markedly reduced.^6^ TAPSE mostly reflects basal RV contractility and is therefore less sensitive to apical RV dysfunction in comparison with RV strain and FAC. TAPSE, FAC, and RV strain were significantly and pathologically reduced in patients with relevant R–L shunt compared with the rest of the M-TEER cohort. Strikingly, the difference in TAPSE is only small (15 vs. 19 mm), and given a usual measurement error of ±1 mm, this does not seem to be a good discriminator. However, the differences for FAC (27% vs. 43%) and RV strain (−13% vs. −22%) are much more pronounced and could better highlight patients at risk for R–L shunt after M-TEER. We would therefore recommend including those measurements in the standard echocardiographic evaluation for patients with greater than moderate tricuspid regurgitation in preparation for M-TEER. The small patient number precludes a definitive statement about an incremental value for including RV strain and FAC; however, our observations suggest that these parameters may help in the identification of high-risk patients for R–L shunt.

sPAP was lower in patients with R–L shunt, which can probably be explained by equalization of pressures between RV and RA due to severe tricuspid regurgitation. This has been described in the literature.^7^ sPAP is therefore not a reliable parameter to identify patients at risk for R–L shunt after M-TEER. RV–PA coupling is an emerging parameter, which reflects the relationship between RV afterload and RV systolic function expressed as the ratio of TAPSE/sPAP. Values of 0.3–0.4 are associated with RV–PA uncoupling.^6^ In our cohort, only one patient had values in this range, which was due to relevant underestimation of pulmonary pressure by sPAP. Echo-derived sPAP does therefore not seem to be reliable in this context. Instead, invasively measured sPAP should be used for evaluation.

If RV function is impaired, pre-procedural right heart catheterization should be performed to obtain invasive haemodynamic measurements. These echocardiographic and invasive measurements are red flags for planning an M-TEER procedure and should prompt organizational steps to ensure readiness for immediate septal occluder implantation.

Patient selection for and timing of M-TEER are highly relevant issues in this patient cohort with older age, high cardiovascular burden, and often relevant frailty, especially in patients with concomitant relevant tricuspid regurgitation as in our patients who required iASD closure. Reducing post-capillary pressure by treating mitral regurgitation reduces the risk of pulmonary oedema and may lead to reduced tricuspid regurgitation. However, if symptoms related to relevant tricuspid regurgitation persist after M-TEER, then T-TEER should be carefully considered.

Secondly, it highlights the importance of measuring RA pressure before or at least during M-TEER as probably the most important factor for R–L shunt post-M-TEER.

Thirdly, in patients with impaired RV function and a RA to LA pressure gradient after M-TEER, oxygenation should be carefully monitored after removal of the steerable catheter. In addition to TEE evaluation of the shunt, peripheral oxygen saturation should be closely monitored, and serial measurements of arterial oxygen saturation and partial pressure of oxygen should be performed. If M-TEER is performed using general anaesthesia, special attention should be given to the communication with the anaesthesiologist, as changes in oxygen saturation may partly be counterbalanced by unnoticed increases in the fraction of inspired oxygen (FiO_2_). We recommend a period of 10 min of observation for deoxygenation. It is not possible to define a precise cut-off for the amount of deoxygenation. We would consider a reduction of peripheral oxygen saturation of ≥3% to be clinically relevant in this often frail patient cohort with a high burden of cardiovascular disease. As a cautionary measure, a guidewire may be placed in the pulmonary vein to ensure quick delivery of a septal occluder if necessary.

We suggest the following clinical approach to identify patients at risk for R–L shunting after M-TEER:

1. If tricuspid regurgitation is at least severe (III°), then right heart dimensions and function (including FAC and strain) should be measured, and invasive right heart pressures should be obtained;
2. careful measurement of LA and RA pressures should be performed; the reduction of LA mean pressure after M-TEER is usually small (1–3 mmHg, see our cohort or Ben-Shoshan *et al.*^8^);
3. if right heart dimensions and function are pathological and RA pressure exceeds LA pressure, then preparations for emergency occluder implantation should be made in preparation for M-TEER;
4. after M-TEER device implantation, the iASD should be carefully evaluated for R–L shunt using TEE, and peripheral oxygen saturation should be closely monitored. From our clinical experience, we suggest a minimum of 10 min for this evaluation, and we consider a drop in peripheral oxygen saturation of ≥3% to be relevant;
5. if there is relevant R–L shunt, then an occluder should be immediately implanted. For sizing of the iASD, we would recommend multi-planar reconstruction using TEE.

### Limitations

The main limitations of these analyses stem from the small number of patients who required iASD closure after M-TEER. This only allows for observation of parameters that are associated with relevant R–L shunt but not for clear identification of risk factors or predictors. Furthermore, this precludes multivariable analysis. The patient cohort is from a single centre and the lack of advanced RV imaging parameters in the comparator population and the retrospective analysis of FAC/strain in only 20 controls reduce the generalizability.

## Conclusion

Relevant R–L shunt causing deoxygenation after M-TEER is a rare but severe complication of this procedure. Careful echocardiographic and haemodynamic evaluation is necessary to identify high-risk patients and take proper organizational measures. Especially reduced RV function was associated with relevant R–L shunt. Close peri-procedural dynamic monitoring of shunt direction and oxygenation for approximately 10 min is crucial to identify the occurrence of R–L shunt. With these cautionary steps, quick detection and treatment with a septal occluder can be assured.

## Data Availability

The presented data may be made available upon reasonable request to the corresponding author.

## Lead author biography

PD Dr med. Michael Wester is a cardiologist and specialist for echocardiography. He started his clinical career in 2018 and has since been part of clinical trials and education of medical students and echocardiography training for cardiologists.
